# Spin and pseudospin solutions to Dirac equation and its thermodynamic properties using hyperbolic Hulthen plus hyperbolic exponential inversely quadratic potential

**DOI:** 10.1038/s41598-020-77756-x

**Published:** 2021-01-13

**Authors:** Ituen B. Okon, E. Omugbe, Akaninyene D. Antia, C. A. Onate, Louis E. Akpabio, O. E. Osafile

**Affiliations:** 1 Theoretical Physics Group, Department of Physics, University of Uyo, Uyo, Nigeria; 2 Department of Physics, Federal University of Petroleum Resources, Effurun, Nigeria; 3 Department of Physical Sciences, Landmark University, Omu-Aran, Nigeria

**Keywords:** Applied mathematics, Pure mathematics, Physics

## Abstract

In this research article, the modified approximation to the centrifugal barrier term is applied to solve an approximate bound state solutions of Dirac equation for spin and pseudospin symmetries with hyperbolic Hulthen plus hyperbolic exponential inversely quadratic potential using parametric Nikiforov–Uvarov method. The energy eigen equation and the unnormalised wave function were presented in closed and compact form. The nonrelativistic energy equation was obtain by applying nonrelativistic limit to the relativistic spin energy eigen equation. Numerical bound state energies were obtained for both the spin symmetry, pseudospin symmetry and the non relativistic energy. The screen parameter in the potential affects the solutions of the spin symmetry and non-relativistic energy in the same manner but in a revised form for the pseudospin symmetry energy equation. In order to ascertain the accuracy of the work, the numerical results obtained was compared to research work of existing literature and the results were found to be in excellent agreement to the existing literature. The partition function and other thermodynamic properties were obtained using the compact form of the nonrelativistic energy equation. The proposed potential model reduces to Hulthen and exponential inversely quadratic potential as special cases. All numerical computations were carried out using Maple 10.0 version and Matlab 9.0 version softwares respectively.

## Introduction

Most of the quantum mechanical systems were investigated through bound state and scattering state solutions of relativistic and nonrelativistic wave equations with considerable potential models. Schrodinger wave equation constitutes the non-relativistic wave equation while Klein–Gordon, Dirac, Duffin–Kemmer–Petiau (DKP) constitute the relativistic wave equation^1,2^. The Dirac equation forms the foundation for the formulation of relativistic quantum mechanics^3^. Dirac equation has wide applications in physical sciences ranging from high energy physics, quantum information, quantum electrodynamics and quantum fluctuations^4–7^. The solutions of Dirac equation is applicable in describing the nuclear shell struture^8–10^. The concept of spin and pseudospin symmetries under Dirac formulation have been used extensively to explain the feature of deformed nuclei and super-deformation thereby establishing and effective shell model coupling scheme^11–13^. Spin symmetry is use for the description of mesons. This arises when the difference of the potential between the repulsive Lorentz vector *V*(*r*) and the attractive Lorentz scalar vector *S*(*r*) is constant; that is, $\Delta = V(r) -S(r)$. However, pseudo symmetry arises when the sum of the potential of the repulsive Lorentz vector potential *V*(*r*) and the attractive Lorentz scalar potential *S*(*r*) is constant; that is $\sum (r) = V(r) + S(r)$^14–16^. The Klein–Gordon equation as Lorentz scalar was use to describe spinless particle while Dirac equation was used for the description of spin-1/2 particles^17–25^. Meanwhile, the relativistic wave equation wether Dirac, Klein–Gordon, and DKP is consider either as mixed vector and scalar potentials or equal vector and scalar potentials^26–30^. In mathematical physics, the Dirac equation with pseudo scalar potential can be handled as a Sturm–Liouville problem while the bound state spectrum of Dirac equation is relevant for the study of Zakharaov–Shabat eigenvalue problem which is applicable in the study of isospectral flow of soliton in plasma^31–34^. The solutions of Dirac equation with various considerable potentials within spin and pseudospin symmetries have been obtained by different authors using different methods such as: factorisation method^35,36^, asymptotic iteration method^37,38^, Laplace transform technique^39^, Supersymmetric quantum mechanics approach^40–42^, path integral formulation^43–45^, Nikiforov–Uvarov method^46,47^ and others.

Both Dirac and Klein–Gordon equations have been studied with different potentials such as: Woods–Saxon, Rosen–Morse, Hulthen, Eckart, Manning Rosen plus Hellmann, Scarf potential and others^48–57^. The investigation of approximate bound state solutions of Dirac equation has been carried out by different authors. Okorie et al.^58^ investigated the solution of Dirac and Schrodinger equation with shifted Tietz–Wei potential where they obtained relativistic and nonrelativistic ro-vibrational energy spectra as well as numerical solutions for different diatomic molecules within the framework of factorisation method. Onyeaju et al.^59^ employed the Perkeris approximation to the centrifugal term to obtain approximate bound state solutions of Dirac equation with some thermodynamic properties for the deformed Hylleraas plus deformed Woods-Saxon potential. Similarly Ikhdair and Hamzavi^60^ studied Dirac bound state solutions of spherically ringed-shape q-deform Woods-Saxon potential for any l-state; where they obtained energy eigenvalue equation and the corresponding two components wave functions within the framework of Nikiforov–Uvarov method. Arda and Sever^3^ studied bound state solutions of Dirac equation for Kratzer potential with pseudo-scalar Coulomb term. Ortakaya et al.^61^ investigated bound state solutions of Dirac equation with Deng–Fan potential including a Coulomb tensor interaction within the framework of asymptotic iteration method where they presented their numerical results for spin and pseudospin limits. A lot of research work have been carried out by Ikot and co-authors on Dirac equation as seen in Refs^62,63^. Other research contributions on Dirac equations were presented in Refs^64–66^.

Some considerable potentials describing physical systems have been modelled as hyperbolic and trigonometric potentials because of their contributions in nuclear and high energy physics. Some authors have reported the applications and usefulness of such potential models. Ikhdair^67^ calculated a rotational and vibrational energies of diatomic molecules in Klein–Gordon equation using hyperbolic scalar and vector potential by means of parametric Nikiforov–Uvarov method. Ikot et al.^68^ studied Schrodinger equation with screen Kratzer potential in the presence of external magnetic and AB flux field using factorisation method. The eigenvalue and the eigenfunction of the system were presented in a close form while magnetisation and magnetic susceptibility were evaluated at zero and finite temperatures as well as thermodynamic properties. Dong et al.^69^ examined quantum information entropies for a squared tangent potential where the calculated position Shannon and momentum entropies that satisfies Beckner, Bialynicki and Mycieslki (BBM) inequality. Okon et al.^70^ obtained eigen solutions to Schrodinger equation with trigonometric Inversely quadratic plus Coulombic hyperbolic potential where they obtained energy eigen equation and normalised wave function using Nikiforov–Uvarov method. Onate^71^ examined bound state solutions of Schrodinger equation with second Poschl–Teller-like potential where he obtained vibrational partition function, mean energy and mean free energy where Poschl–Teller-like potential was expressed in the form of hyperbolic cosh and sinh. Okorie et al.^72^ obtained the ro-vibrational energy spectra of the improved deformed exponential-type potential using Greene–Aldrich approximation scheme and coordinate transformation where they obtained vibrational partition function and other thermodynamic properties using poisson summation formular.

In this research article, we solve bound state solutions of Dirac equation with equal scalar and vector potential using hyperbolic Hulthen plus hyperbolic Yukawa inversely quadratic potential under spin and pseudospin symmetries using the parametric Nikiforov–Uvarov method. This work is also extended to study some thermodynamic properties using the nonrelativistic energy eigen equation. This article is divided into seven sections. “Introduction” gives the brief introduction of the article. The basic theory of Dirac equation is presented in “Dirac equation for spin and pseudospin symmetry” section. Parametric Nikiforov–Uvarov method is presented in “The parametric Nikiforov–Uvarov (NU) method” section while the analytical solution of Dirac equation is presented in “Solution of Dirac equation with the proposed potential” section. The numerical results for both spin and pseudospin symmetries are presented in “Numerical results” section. Thermodynamic properties is presented in “Thermodynamic properties for the potential model” section while the discussion of numerical results and tables are done in “Discussion” section. The research article is concluded in “Conclusion” section.

## Dirac equation for spin and pseudospin symmetry

The Dirac equation^8^ for spin-1/2 particles moving in a field of attractive scalar potential *S*(*r*) and repulsive vector potential *V*(*r*) is given as

1 $$\begin{aligned}{}[\alpha \cdot p +\beta (M+S(r))]{\psi (r)}= [E-V(r)]{\psi (r)} \end{aligned}$$

where *E* is the relativistic energy, *M* is the mass of a single particle, $p=-i{\Delta }$ is the momentum operator, while $\alpha$ and $\beta$ are the $4\times 4$ Pauli matrices defined as

2 $$\begin{aligned} \alpha = \begin{pmatrix}0&{}\quad \sigma _i\\ \sigma _i&{}\quad 0\end{pmatrix},\,\beta = \begin{pmatrix}I&{}\quad 0\\ 0&{}\quad -I\end{pmatrix} \end{aligned}$$

Here *I* is the $2\times 2$ unitary matrix and $\sigma _i$ are the three vector spin matrices given as

3 $$\begin{aligned} \sigma _1=\begin{pmatrix}0&{}\quad 1\\ 1&{}\quad 0\end{pmatrix},\, \sigma _2=\begin{pmatrix}0&{}\quad -i\\ i&{}\quad 0\end{pmatrix},\, \sigma _3=\begin{pmatrix}1&{}\quad 0\\ 0&{}\quad 1\end{pmatrix} \end{aligned}$$

The total angular momentum *J* and the spin orbit operator $\hat{K}=-\beta (\alpha \cdot L+1)$ of a particle commutes with the Dirac Hamiltonian in a central field where *L* is the orbital angular momentum of the spherical nucleons. The eigenvalues of the spin-orbit coupling operator $\hat{K}$ are $\kappa =-(j+\frac{1}{2})<0$, $j=(l-\frac{1}{2})$ for unaligned spin and $\kappa =(j+\frac{1}{2})>0$, $j=(l+\frac{1}{2})$ for aligned spin respectively. Thus, the spinor wave function can be classified according to the angular momentum *j*, the spin orbit quantum number $\kappa$ and the radial quantum number *n*. The wave function can then be written as $\kappa$ and the radial quantum number *n*. The wave function can then be written as

4 $$\begin{aligned} \psi (\vec {r}) =\begin{pmatrix}\xi (\vec {r})\\ \phi (\vec {r})\end{pmatrix} =\frac{1}{r}\begin{pmatrix}\ F_{nk}({r})\ Y_{jm}^{l}(\theta , \varphi )\\ \ iG_{nk}({r})\ Y_{jm}^{\tilde{l}}(\theta , \varphi )\end{pmatrix} \end{aligned}$$

where $F_{nk}$ and $G_{nk}$ are the upper and lower radial wave functions respectively. $Y_{jm}^{l}(\theta , \varphi )$ and $Y_{jm}^{\tilde{l}}(\theta , \varphi )$ are the spin and pseudospin spherical harmonics respectively. *m* is the projection of angular momentum on the z-axis. Substituting Eqs. (1)–(4) and by making use of the following relations

5 $$\begin{aligned} (\hat{\sigma }\cdot \vec {A})\cdot (\hat{\sigma }\cdot \vec {B})& {}=  \vec {A}\cdot \vec {B}+i\hat{\sigma }\cdot (\vec {A}\times \vec {B}) \end{aligned}$$

6 $$\begin{aligned} \hat{\sigma }\cdot \hat{p}& {}=  \hat{\sigma }\cdot \hat{r}\left( \hat{r}\cdot \hat{p}+\frac{i\hat{\sigma }\tilde{l}}{r}\right) \end{aligned}$$

Together with the additional properties

7 $$\begin{aligned} \left. \begin{array}{l} (\hat{\sigma }\cdot \vec {L})\ Y_{jm}^{\tilde{l}}(\theta , \varphi ) =(\kappa -1)\ Y_{jm}^{\tilde{l}}(\theta , \varphi ) \\ (\hat{\sigma }\cdot \vec {L})\ Y_{jm}^{l}(\theta , \varphi ) =(\kappa -1)\ Y_{jm}^{l}(\theta , \varphi ) \\ (\hat{\sigma }\cdot \hat{r})\ Y_{jm}^{\tilde{l}}(\theta , \varphi )=-\ Y_{jm}^{l}(\theta , \varphi ) \\ (\hat{\sigma }\cdot \hat{r})\ Y_{jm}^{l}(\theta , \varphi )=-\ Y_{jm}^{\tilde{l}}(\theta , \varphi ) \end{array}\right\} \end{aligned}$$

result into two coupled differential equations whose solutions constitutes the upper $$ F_{nk} $$ and the lower $$ G_{nk}$$ as follows:

8 $$\begin{aligned} \left[ \frac{d}{dr}+\frac{\kappa }{r}-U(r)\right] F_{nk}(r)=(M+\ E_{nk}-\Delta (r))\ G_{nk}(r) \end{aligned}$$

9 $$\begin{aligned} \left[ \frac{d}{dr}-\frac{\kappa }{r}+U(r)\right] G_{nk}(r)=\left( M-\ E_{nk}+\sum (r)\right) \ F_{nk}(r) \end{aligned}$$

where

10 $$\begin{aligned} \Delta (r)=V(r)-S(r), \,\,\,\sum (r)=V(r)+ S(r) \end{aligned}$$

By eliminating $$ F_{nk}(r)$$ and $$ G_{nk}(r)$$ from Eqs. (8) and (9), the following Schrodinger-like differential equations for the upper and lower radial spinor components are obtained as follow:

11 $$\begin{aligned}&\left[ \frac{d^{2}}{dr}-\frac{\kappa (\kappa +1)}{r^{2}}+\frac{2\kappa U(r)}{r}-\frac{dU(r)}{dr}-U^{2}(r)\right] F_{nk}(r) +\frac{\frac{d\Delta (r)}{dr}}{M+\ E_{nk}-\Delta (r)}\left[ \frac{d}{dr}+\frac{\kappa }{r}-U(r)\right] F_{nk}(r) \nonumber \\&\quad =\left[ (M+\ E_{nk}-\Delta (r))(M-\ E_{nk}-\sum (r))\right] F_{nk}(r) \end{aligned}$$

12 $$\begin{aligned}&\left[ \frac{d^{2}}{dr}-\frac{\kappa (\kappa -1)}{r^{2}}+\frac{2\kappa U(r)}{r}+\frac{dU(r)}{dr}-U^{2}(r)\right] G_{nk}(r) +\frac{\frac{d\sum (r)}{dr}}{M+\ E_{nk}-\sum (r)}\left[ \frac{d}{dr}-\frac{\kappa }{r}+U(r)\right] G_{nk}(r) \nonumber \\&\quad =\left[ (M+\ E_{nk}-\Delta (r))(M-\ E_{nk}-\sum (r))\right] G_{nk}(r) \end{aligned}$$

where $\kappa (\kappa +1)=\tilde{l}(\tilde{l}+1)$ and $\kappa (\kappa -1)= l(l+1)$. The relationship between the quantum number $\kappa$ and the quantum numbers for spin *l* and pseudospin $\tilde{l}$ symmetries are

13 $$\begin{aligned} \kappa& {}=  \left\{ \begin{array}{lll} -(l+1) = - \left( j+\frac{1}{2}\right) \left( s_{\frac{1}{2}},\ p_{\frac{3}{2}}\right) \\ j = l+\frac{1}{2}; \,\, \text {aligned spin}\,\, (\kappa <0)\\ (\tilde{l}+1) = \left( j+\frac{1}{2}\right) \left( d_{\frac{3}{2}},\ d_{\frac{5}{2}}\right) \\ l = l-\frac{1}{2}; \,\,\text {unaligned spin}\,\, (\kappa >0)\\ \end{array}\right. \end{aligned}$$

14 $$\begin{aligned} \kappa& {}=  \left\{ \begin{array}{lll} -\tilde{l} = - \left( j+\frac{1}{2}\right) \left( s_{\frac{1}{2}},\ p_{\frac{3}{2}} \right) \\ j = l-\frac{1}{2}; \,\, \text {aligned spin}\,\, (\kappa <0)\\ (\tilde{l}+1) = \left( j+\frac{1}{2}\right) \left( d_{\frac{3}{2}},\ d_{\frac{5}{2}}\right) \\ j = \tilde{l}-\frac{1}{2}; \,\,\text {unaligned spin}\,\, (\kappa >0)\\ \end{array}\right. . \end{aligned}$$

However, for spin symmetry limit, $\Delta (r)$ is a constant that is $c_{s}=\frac{d\Delta (r)}{dr}=0$ whereas for pseudospin symmetry limit $\sum (r)$ is a constant such that $c_{ps}=\frac{d\sum (r)}{dr}=0$

## The parametric Nikiforov–Uvarov (NU) method

The NU method is based on reducing second order linear differential equation to a generalised equation of hyper-geometric type and provides exact solutions in terms of special orthogonal functions like Jacobi and Laguerre as well as corresponding energy eigenvalues^73–78^. The standard differential equation for parametric NU method according to Tezcan and Sever^79^ is given as

15 $$\begin{aligned} \psi ''(s)+\frac{(c_{1} -c_{2} s)}{s(1-c_{3} s)} \psi '(s)+\frac{1}{s^{2} (1-c_{3} s)^{2} } \left[ -\Omega _{1} s^{2} +\Omega _{2} s-\Omega _{3} \right] \psi (s)=0 \end{aligned}$$

The parametric constants are obtained as follows

16 $$\begin{aligned} \left. \begin{array}{l} {c_{4} =\frac{1}{2} \left( 1-c_{1} \right) ;\, \, \, c_{5} =\frac{1}{2} \left( c_{2} -c_{3} \right) ;\, \, c_{6} =c_{5}^{2} +\epsilon _{1} } \\ {c_{7} =2c_{4} c_{5} -\Omega _{2} ;\, \, c_{8} =c_{4}^{2} +\Omega _{3} ;\, \, \, c_{9} =c_{3} c_{7} +c_{3}^{2} c_{8} +c_{6} } \\ {c_{10} =c_{1} +2c_{4} +2\sqrt{c_{8} } ;\, \, \, c_{11} =c_{2} -2c_{5} +2\left( \sqrt{c_{9} } +c_{3} \sqrt{c_{8} } \right) } \\ {c_{12} =c_{4} +\sqrt{c_{8} } ;\, \, c_{13} =c_{5} -\left( \sqrt{c_{9} } +c_{3} \sqrt{c_{8} } \right) } \end{array}\right\} . \end{aligned}$$

The condition for energy equation is given as

17 $$\begin{aligned} c_{2}^{} n-\left( 2n+1\right) c_{5} \left( 2n+1\right) \left( \sqrt{c_{9} } +c_{3} \sqrt{c_{8} } \right) +n\left( n-1\right) c_{3} +c_{7}+ 2c_{3} c_{8} +2\sqrt{c_{8} c_{9} } =0 \end{aligned}$$

The corresponding total wave function is then given as

18 $$\begin{aligned} \Psi (s)=N_{nl} s^{c_{12} } \left( 1-c_{3} s\right) ^{-c_{12} -\frac{c_{11} }{c_{3} } } P_{n}^{\left( c_{10} -1,\, \, \frac{c_{11} }{c_{3} } -c_{10} -1\right) } \left( 1-2c_{3} s\right) . \end{aligned}$$

## Solution of Dirac equation with the proposed potential

Dirac equation^8^ with equal scalar and vector potential without tensor interaction is given as

19 $$\begin{aligned} \left[ \frac{d^{2}}{dr^{2}}-\frac{k(k+1)}{r^{2}}-2(E_{nl}+M)V(r)+(E_{nl}^{2}-M^{2})\right] F_{nk}=0 \end{aligned}$$

Equation (19) can further be express as

20 $$\begin{aligned} \frac{d^{2}F_{nk}}{dr^{2}}+\left[ -\frac{k(k+1)}{r^{2}}-2\gamma V(r)+\beta ^{2}\right] F_{nk}=0 \end{aligned}$$

where

21 $$\begin{aligned} \gamma = (E_{nl}^{2}-M^{2}), \beta = (E_{nl}^{2}-M^{2}). \end{aligned}$$

The hyperbolic Hulthen plus hyperbolic exponential inversely quadratic potential is given as

22 $$\begin{aligned} V(r) = \left[ \frac{v_{1}e^{-\frac{r}{b}}\cosh \omega }{1- e^{-\frac{r}{b}}\cosh \omega } - \frac{A e^{-\frac{r}{b}}\cosh \omega }{r^{2}}\right] \end{aligned}$$

where $v_{1}$ is the potential depth, *A* is a real constant parameter, *b* is the screening parameter representing the strength of the potential and $\omega$ is the optimising parameter. The potential of Eq. (22) can be express as

23 $$\begin{aligned} V(r)=\left[ \frac{v_{1}q e^{-\upsilon r}}{1-q e^{-\upsilon r}}-\frac{Aq e^{-\upsilon r}}{r^{2}}\right] \end{aligned}$$

where $\upsilon =\frac{1}{b}$, $q=\cosh {\omega }$. The standard Greene–Aldrich approximation to the centrifugal term suitable for the propose potential is given as

24 $$\begin{aligned} \frac{1}{r^2} = \frac{\upsilon ^2qe^{-\upsilon r}}{(1 - qe^{-\upsilon r})} \end{aligned}$$

Substituting (23) and (24) into (20) and simplifying gives

25 $$\begin{aligned} \frac{d^{2}F_{nk}}{dr^{2}}+\left[ -\frac{k(k+1)\upsilon ^{2}qe^{-\upsilon r}}{(1-q e^{-\upsilon r})^{2}}+\frac{2\gamma v_{1}qe^{-\upsilon r}}{(1-q e^{-\upsilon r})}+\frac{2A\gamma \upsilon ^{2}q^{2}e^{-2\upsilon r}}{(1-q e^{-\upsilon r})^{2}}+\beta ^{2}\right] F_{nk}=0 \end{aligned}$$

Let $s=e^{-\upsilon r}$, then (25) reduces to

26 $$\begin{aligned}&\frac{d^{2}F_{nk}}{dr^{2}} +\frac{(1-qs)}{s(1-qs)}\frac{dF_{nk}}{ds} +\frac{1}{s^{2}(1-s)^{2}}\left[ -\left( \frac{2\gamma v_{1}q^{2} }{\upsilon ^{2}}-2A\gamma q^{2}-\frac{q^{2}\beta ^{2}}{\upsilon ^{2}}\right) s^{2}\right. \nonumber \\&\quad +\,\left. \left( \kappa (\kappa +1)q-\frac{2q\beta ^{2} }{\upsilon ^{2}}+\frac{2\gamma v_{1}q }{\upsilon ^{2}}\right) s -\left( -\frac{\beta ^{2}}{\upsilon ^{2}}\right) \right] F_{nk} = 0 \end{aligned}$$

Comparing (26)–(15), the followings are obtain

27 $$\begin{aligned} \left[ \Omega _{1}=\left( \frac{2\gamma v_{1}q^{2} }{\upsilon ^{2}}-2A\gamma q^{2}-\frac{q^{2}\beta ^{2}}{\upsilon ^{2}}\right) ,\,\,\Omega _{2}= \left( \kappa (\kappa +1)q-\frac{2q\beta ^{2} }{\upsilon ^{2}}+\frac{2\gamma v_{1}q }{\upsilon ^{2}}\right) ,\, \Omega _{3}=\left( -\frac{\beta ^{2}}{\upsilon ^{2}}\right) \right] \end{aligned}$$

Using Eq. (16), the following parametric constants are obtained as follows:

28 $$\begin{aligned} \left. \begin{array}{l} c_1 =1,\,\, c_{2} = c_{3} = q;\,\, c_{4} =0;\, \, \, c_{5} = -\frac{q}{2} ;\, \, c_{6} =\frac{q^{2}}{4}+ \frac{2\gamma v_{1}q^{2} }{\upsilon ^{2}}-2A\gamma q^{2}-\frac{q^{2}\beta ^{2}}{\upsilon ^{2}} \\ c_{7} =-\kappa (\kappa +1)q+\frac{2q\beta ^{2} }{\upsilon ^{2}}-\frac{2\gamma v_{1}q }{\upsilon ^{2}} ;\, \, c_{8} =-\frac{\beta ^{2}}{\upsilon ^{2}} ;\, \, \, c_{9} =\kappa (\kappa +1)q^{2} +\frac{q^{2}}{4}-2\gamma q^{2}A\\ c_{10} =1+2\sqrt{-\frac{\beta ^{2}}{\upsilon ^{2}}} ;\, \, \, c_{11} =2q+2\sqrt{4\kappa (\kappa +1)q^{2}-8A\gamma q^{2}+q^{2}}+2q\sqrt{-\beta ^{2}b^{2}} \\ c_{12} =\sqrt{-\beta ^{2}b^{2}} ;\, \, c_{13} =-\frac{q}{2}-\left[ \frac{1}{2}\sqrt{4\kappa (\kappa +1)q^{2}-8A\gamma q^{2}+q^{2}}+2q\sqrt{-\beta ^{2}b^{2}}\right] \end{array}\right\} . \end{aligned}$$

Using Eq. (17) with some algebraic simplification, we have

29 $$\begin{aligned} \beta ^{2}=-\frac{1}{b^{2}}\left[ \frac{(n^{2}+n+\frac{1}{2})q +q(n+\frac{1}{2})\sqrt{(2\kappa +1)^{2}-8A\gamma }+q\kappa (\kappa +1)-2q\gamma v_{1}b^{2}}{(2n+1)q+q\sqrt{(2\kappa +1)^{2}-8A\gamma }}\right] \end{aligned}$$

substituting (21)–(29) gives the relativistic spin energy equation as

30 $$\begin{aligned} (E_{nk}^{s}-M)(E_{nk}^{s}+M)=-\frac{1}{b^{2}} \left[ \frac{\begin{array}{l}(n^{2}+n+\frac{1}{2})q +q(n+\frac{1}{2})\sqrt{(2\kappa +1)^{2}-8A(E_{nk}^{s}+M)} \\ \quad +\, q\kappa (\kappa +1) - 2q(E_{nk}^{s}+M) v_{1}b^{2}\end{array}}{(2n+1)q+q\sqrt{(2\kappa +1)^{2}-8A(E_{nk}^{s}+M)}}\right] ^{2}. \end{aligned}$$

Equation (30) can also be express as

31 $$\begin{aligned} (E_{nk}^{s}-M)(E_{nk}^{s}+M)=-\frac{1}{b^{2}} \left[ \frac{\begin{array}{l}\left( n^{2}+n+\frac{1}{2}\right) \cosh \omega \\  \quad+\, \left( n+\frac{1}{2}\right) \sqrt{(2\kappa +1)^{2}-8A(E_{nk}^{s}+M)}\cosh \omega \\ \quad+\, \kappa (\kappa +1)\cosh \omega -2(E_{nk}^{s}+M) v_{1}b^{2}\cosh \omega \end{array}}{(2n+1)\cosh \omega +\sqrt{(2\kappa +1)^{2}-8A(E_{nk}^{s}+M)}\cosh \omega }\right] ^{2}. \end{aligned}$$

In order to obtain the solution for the pseudospin symmetry, then the condition for pseudospin symmetry limit discussed in “Dirac equation for spin and pseudospin symmetry” section is fully applied such that $\gamma =E_{nk}-M-c_{ps}$, $\beta =(M+E_{nk}-c_{ps})$, $c_{ps}=0$. Following the same steps to obtain the energy equation for the spin symmetry, the energy equation for pseudospin symmetry limit is then given as

32 $$\begin{aligned} (M+E_{nk}^{ps})(M-E_{nk}^{ps})=-\frac{1}{b^{2}} \left[ \frac{\begin{array}{l} (n^{2}+n+\frac{1}{2})q +q(n+\frac{1}{2})\sqrt{(1-2\kappa )^{2}-8A(E_{nk}^{s}+M)}\\ \quad+\,q\kappa (\kappa -1)-2q(E_{nk}^{ps}-M) v_{1}b^{2}\end{array}}{(2n+1)q+q\sqrt{(1-2\kappa )^{2}-8A(E_{nk}^{ps}-M)}}\right] ^{2}. \end{aligned}$$

Using Eq. (8), the upper component of the wave function is given as

33 $$\begin{aligned} \Psi _{nl}& {}=  N_{nl}(e^{-\frac{r}{b}}\cosh \omega )^{ \sqrt{-\beta ^{2}b^{2}}}(1-e^{-\frac{r}{b}}\cosh \omega )^\eta \nonumber \\&\quad \times\, P_n^{\left[ \sqrt{-\beta ^{2}b^{2}}, \,\,\,2\cosh \omega + 2\sqrt{4\kappa (\kappa +1)(\cosh \omega )^2 -8A\gamma (\cosh \omega )^{2}+(\cosh \omega )^2} + 2\cosh \omega \sqrt{-\beta ^{2}b^{2}} - 2\sqrt{-\beta ^{2}b^{2}} - 2\right] }\nonumber \\&\quad \times\,(1-2e^{-\frac{r}{b}}\cosh \omega ) \end{aligned}$$

where

$$\begin{aligned} \eta& {}=  -\sqrt{-\beta ^{2}b^{2}}-\left[ 2\cosh \omega + 2\sqrt{4\kappa (\kappa +1)(\cosh \omega )^2 -8A\gamma (\cosh \omega )^{2}+(\cosh \omega )^2}\right. \\&\quad \left. +\, 2\cosh \omega \sqrt{-\beta ^{2}b^{2}}\right] . \end{aligned}$$

The lower component of the Dirac spinor can be evaluated using the equation

34 $$\begin{aligned} G_{nk}(r)=\frac{1}{M+E_{nk}}\left[ \frac{d}{dr}+\frac{k}{r}\right] F_{nk}(r) \end{aligned}$$

where $$E_{nk}\ne -M+ c_{s}$$

### Non-relativistic solution

The non-relativistic(NR) solution of the spin symmetry leads to the Schrodinger-like solution. This is obtain by applying non-relativistic limit to the relativistic spin energy equation (30). The non relativistic transformation equations are $M+E_{nk}=\frac{2\mu }{\hbar ^{2}}$, $M-E_{nk}=-E_{nl}$ as $\kappa \rightarrow l$. Substituting above condition into (31) and simplifying gives the nonrelativistic energy eigen equation as

35 $$\begin{aligned} E_{nl}=-\frac{\hbar ^{2}}{2\mu b^{2}}\left[ \frac{q(n^{2}+n+\frac{1}{2})+q(n+\frac{1}{2})\sqrt{(2l+1)^2-\frac{8\mu A}{\hbar ^{2}}}+ql(l+1)-\frac{2q\mu v_{1}b^{2}}{\hbar ^{2}}}{(2n+1)q+q\sqrt{(2l+1)^2-\frac{8\mu A}{\hbar ^{2}}}}\right] ^{2}. \end{aligned}$$

### Deduction for special cases

- Hulthen potential: Substituting $A=0, q=1$ into (23), the potential reduces to Hulthen potential.
  36 $$\begin{aligned} V(r)=-\frac{v_{1}e^{-\frac{r}{b}}}{(1-e^{-\frac{r}{b}})} \end{aligned}$$
  The resulting energy equation is given as
  37 $$\begin{aligned} E_{nl}=-\frac{\hbar ^{2}}{2\mu b^{2}}\left[ \frac{(1+n+1)}{2}-\frac{\frac{2\mu v_{1}b^{2}}{\hbar ^{2}}}{2(1+n+l)}\right] ^{2} \end{aligned}$$
  However, for the purpose of comparison of the Hulthen potential to an existing literature, let $v_{1}=\frac{ze^{2}}{b}$ where $z=e=1$ in atomic mass unit. Substituting $v_{1}$ into Eq. (37) gives the energy equation of the Hulthen potential as
  38 $$\begin{aligned} E_{nl}=-\frac{\hbar ^{2}}{2\mu b^{2}}\left[ \frac{(1+n+1)}{2}-\frac{\frac{2\mu b}{\hbar ^{2}}}{2(1+n+l)}\right] ^{2}. \end{aligned}$$
- Exponential inversely quadratic potential: When $v_{1}=0$, and $q=1$, the potential reduces to exponential inversely quadratic potential
  39 $$\begin{aligned} V(r)=\frac{-Ae^{-\frac{r}{b}}}{r^{2}} \end{aligned}$$
  The resulting energy equation is given as
  40 $$\begin{aligned} E_{nl}=-\frac{\hbar ^{2}}{2\mu b^{2}}\left[ \frac{(n^{2}+n+\frac{1}{2})+(n+\frac{1}{2})\sqrt{(2l+1)^2-\frac{8\mu A}{\hbar ^{2}}}+l(l+1)}{(2n+1)+\sqrt{(2l+1)^2-\frac{8\mu A}{\hbar ^{2}}}}\right] ^{2}. \end{aligned}$$

## Thermodynamic properties for the potential model

The thermodynamic properties for the potential model is calculated using Eq. (35) for $q=1$. The thermodynamic properties for a quantum mechanical systems can be obtained from the exact partition function given by

41 $$\begin{aligned} Z(\beta )=\sum _{n=0}^{\lambda } e^{-\beta E_{n}} \end{aligned}$$

where $\lambda$ is an upper bound of the vibrational quantum number obtained from the numerical solution of $\frac{dE_{n}}{dn}=0$, $\beta =\frac{1}{kT}$ where *K* and *T* are Boltzmann constant and absolute temperature respectively. In the classical limit, the summation in (41) can be replaced with the integral:

42 $$\begin{aligned} Z(\beta ) = \int _{0}^{\lambda } e^{-\beta E_{n}}dn \end{aligned}$$

The energy equation of Eq. (35) can be simplified to

43 $$\begin{aligned} E_{nl}=-Q_{1}\left[ (n+\Delta )+\frac{Q_{2}}{(n+\Delta )}\right] ^{2} \end{aligned}$$

where

44 $$\begin{aligned} Q_{1}=\frac{\hbar ^{2}}{8\mu b^{2}},\,\ Q_{2}=\frac{2\mu }{\hbar ^{2}}(A-V_{1}b^{2}),\,\ \Delta =\frac{1}{2}+\frac{1}{2}\sqrt{\left( l+\frac{1}{2}\right) ^2-\frac{2\mu A}{\hbar ^{2}}} \end{aligned}$$

The maximum vibrational quantum number is given by $\lambda =-\Delta +\sqrt{Q_{2}}$. The energy equation (43) can then be express in the form

45 $$\begin{aligned} E_{nl}=-\left[ Q_{1}\rho ^{2}+\frac{Q_{1}Q_{2}^{2}}{\rho ^{2}}\right] -2Q_{1}Q_{2} ,\,\, \rho = n+\Delta \end{aligned}$$

Hence, the partition function Eq. (42) can be express in the classical limit as

46 $$\begin{aligned} Z(\beta )=e^{2\beta Q_{1} Q_{2}}\int _{0}^{\lambda }e^{\left( Q_{1}\rho ^{2}+\frac{Q_{1}Q_{2}^{2}}{\rho ^{2}}\right) }d\rho \end{aligned}$$

Equation (46) is integrated using MAPLE package. Hence, the integral equation (46) which is the partition function is given as

47 $$\begin{aligned} Z(\beta )= e^{2\beta Q_{1}Q_{2}}\sqrt{\pi }\left[ \frac{\begin{array}{l} e^{2\beta Q_{1}Q_{2}}erf \left(\sqrt{-\beta Q_{1}}\lambda +\frac{Q_{2}\sqrt{-\beta Q_{1}}}{\lambda }\right)\\ \quad+\, e^{-2\beta Q_{1}Q_{2}}erf \left(\sqrt{-\beta Q_{1}}\lambda -\frac{Q_{2}\sqrt{-\beta Q_{1}}}{\lambda }\right)\end{array}}{4\sqrt{-\beta Q_{1}}}\right] \end{aligned}$$

Using the partition function (47), other thermodynamic properties are obtain as follows

- Vibrational mean energy:
  48 $$\begin{aligned} U(\beta )=-\frac{\partial \ln Z(\beta )}{\partial \beta }= -\frac{1}{2}\left[ \frac{\begin{array}{l} 8Q_{1}Q_{2}\sqrt{\pi }\beta \lambda e^{2\beta Q_{1}Q_{2}}erf(\chi _{1}) + 2\lambda ^{2}\sqrt{-\beta Q_{1}}\chi _{3}+2Q_{2}\sqrt{-\beta Q_{1}}\chi _{3}\\ \quad+\,2\lambda ^{2}\sqrt{-\beta Q_{1}}\chi _{4}-2Q_{2}\sqrt{-\beta Q_{1}}\chi _{4} - \sqrt{\pi }\lambda e^{2\beta Q_{1}Q_{2}}erf(\chi _{1}) \ \sqrt{\pi }\lambda e^{-2\beta Q_{1}Q_{2}}erf(\chi _{2})\end{array}}{\sqrt{\pi }\beta \lambda \left[ e^{2\beta Q_{1}Q_{2}}erf(\chi _{1})+e^{-2\beta Q_{1}Q_{2}}erf(\chi _{2})\right] }\right] \end{aligned}$$
  where
  49 $$\begin{aligned} \left. \begin{array}{l} \chi _{1}= \left(\frac{\sqrt{-\beta Q_{1}}(\lambda ^{2}+Q_{2})}{\lambda }\right) \\ \chi _{2} = \left(\frac{\sqrt{-\beta Q_{1}}(\lambda ^{2}-Q_{2})}{\lambda }\right)\\ \chi _{3} =e^{\frac{\beta Q_{1}(4Q_{2}\lambda ^{2}+\lambda ^{4}+Q_{2}^{2})}{\lambda ^{2}}} \\ \chi _{4} =e^{\frac{\beta Q_{1}(-4Q_{2}\lambda ^{2}+\lambda ^{4}+Q_{2}^{2})}{\lambda ^{2}}} \end{array}\right\} . \end{aligned}$$
- Vibrational specific heat capacity:
  50 $$\begin{aligned} C(\beta )=k\beta ^{2}\left( \frac{\partial ^{2}\ln Z(\beta )}{\partial \beta ^{2}}\right) =\frac{1}{2}k\left[ \frac{\chi _{9}+\chi _{10}+\chi _{11}+\chi _{12}+\chi _{13}}{\sqrt{-\beta Q_{1}}\pi \lambda ^{3}\left[ e^{2\beta Q_{1}Q_{2}}erf{(\chi _{1})}+e^{-2\beta Q_{1}Q_{2}}erf{(\chi _{2})}\right] ^{2}}\right] \end{aligned}$$
  where
  51 $$\begin{aligned}\left. \begin{array}{lll} \chi _{5} &=e^{\frac{\beta Q_{1}(6Q_{2}\lambda ^{2}+\lambda ^{4}+Q_{2}^{2})}{\lambda ^{2}}}\\ \chi _{6} &= e^{\frac{\beta Q_{1}(-6Q_{2}\lambda ^{2}+\lambda ^{4}+Q_{2}^{2})}{\lambda ^{2}}} \\ \chi _{7} &= e^{\frac{2\beta Q_{1}(4Q_{2}\lambda ^{2}+\lambda ^{4}+Q_{2}^{2})}{\lambda ^{2}}}\\ \chi _{8} &= e^{\frac{2\beta Q_{1}(-4Q_{2}\lambda ^{2}+\lambda ^{4}+Q_{2}^{2})}{\lambda ^{2}}}\\ \chi _{9} &= -\beta Q_{1}Q_{2}\lambda ^{2}\sqrt{\pi } e^{\frac{\beta Q_{1}(\lambda ^{2}+Q_{2})^{2}}{\lambda ^{2}}}erf(\chi _{2})+\beta Q_{1}Q_{2}\lambda ^{2}\sqrt{\pi } e^{\frac{\beta Q_{1}(\lambda ^{2}-Q_{2})^{2}}{\lambda ^{2}}}erf(\chi _{1})\\ & \quad -2Q_{1}\lambda ^{5}\beta \sqrt{-\beta Q_{1}}\chi _{8}\\ & \quad -\lambda ^{3}\pi \sqrt{-\beta Q_{1}}e^{4\beta Q_{1} Q_{2}}erf(\chi _{1})^{2}-\lambda ^{3}\pi \sqrt{-\beta Q_{1}}e^{-4\beta Q_{1} Q_{2}}erf(\chi _{1})^{2}\\  &\quad  -4 Q_{1}Q_{2}\lambda ^{3}\beta \sqrt{-\beta Q_{1}}\chi _{3}+4 Q_{1}Q_{2}\lambda ^{3}\beta \sqrt{-\beta Q_{1}}\chi  _{8}-2Q_{1}Q_{2}^{2}\lambda \beta \sqrt{-\beta Q_{1}}\chi _{7};\\ \chi _{10} &= 4Q_{1}Q_{2}^{2}\lambda \beta \sqrt{-\beta Q_{1}}e^{\frac{2\beta Q_{1}(\lambda ^{4}+Q_{2}^{2})}{\lambda ^{2}}}-2Q_{1}Q_{2}^{2}\lambda \beta \sqrt{-\beta Q_{1}}\chi _{8}\\ & \quad -\beta Q_{1}\lambda ^{4}\sqrt{\pi }e^{\frac{\beta Q_{1}(\lambda ^{2}+Q_{2})^{2}}{\lambda ^{2}}}erf(\chi _{2})-\beta Q_{1}\lambda ^{4}\sqrt{\pi }e^{\frac{\beta Q_{1}(\lambda  ^{2}-Q_{2})^{2}}{\lambda ^{2}}}erf(\chi _{1})\\ & \quad +2Q_{1}^{2}\sqrt{\pi }\beta ^{2}\lambda ^{6}e^{\frac{\beta Q_{1}(\lambda ^2+Q_{2}^{2})}{\lambda ^{2}}}. \end{array}\right\} \end{aligned}$$
  52 $$\begin{aligned}\left. \begin{array}{lll}\chi _{11} &= 2Q_{1}^{2}Q_{2}^{3}\sqrt{\pi }\beta ^{2}e^{\frac{\beta Q_{1}(\lambda ^{2}+Q_{2})^{2}}{\lambda ^{2}}}erf(\chi _{2})+2Q_{1}^{2}\lambda ^{6}e^{\frac{\beta Q_{1}(\lambda ^{2}+Q_{2})^{2}}{\lambda ^{2}}}erf(\chi _{1})\\ & \quad  -2Q_{1}^{2}Q_{2}^{3}\beta ^{2}e^{\frac{\beta Q_{1}(\lambda ^{2}+Q_{2})^{2}}{\lambda ^{2}}}erf(\chi _{1})+2Q_{1}^{2}\sqrt{\pi }\beta ^{2}\chi _{5}\lambda ^{6}erf(\chi _{1})+2Q_{1}^{2}\sqrt{\pi }\beta ^{2}\chi _{5}\lambda ^{6}erf(\chi _{2})\\ & \quad  - 2Q_{1}^{2}Q_{2}^{3}\sqrt{\pi }\beta ^{2}\chi _{6} erf(\chi _{2});\\ \chi _{12} &= -2\sqrt{-\beta Q_{1}}\lambda ^{3}\pi erf(\chi _{1}) erf(\chi _{2})-2Q_{1}\lambda ^{5}\beta \sqrt{-\beta Q_{1}} erf(\chi _{3})\\ & \quad -4 Q_{1}\lambda ^{5}\beta \sqrt{-\beta Q_{1}}e^{\frac{2\beta Q_{1}(\lambda ^{4}+Q_{2}^{2})}{\lambda ^{2}}}-\beta Q_{1}Q_{2}\lambda ^{2}\sqrt{\pi }\chi _{6}erf(\chi _{2})\\  &\quad +6Q_{1}^{2}\sqrt{\pi }\beta ^{2}\lambda ^{4}\chi _{5}Q_{2}erf (\chi _{1}) + 6Q_{1}^{2}\sqrt{\pi }\beta ^{2}\lambda ^{2}\chi _{5}Q_{2}^{2}erf (\chi _{1})\\ \chi _{13} &= -6Q_{1}^{2}\sqrt{\pi }\beta ^{2}\chi _{6}\lambda ^4 Q_{2}erf(\chi _{2})-32Q_{1}^{2}\pi \beta ^{2}\lambda ^{3}Q_{2}^{2}erf(\chi _{1})\sqrt{-\beta Q_{1}} erf(\chi _{2})\\  &\quad + 6Q_{1}^{2}\sqrt{\pi }\beta ^{2}\lambda ^{2}\chi _{6}Q_{2}^{2}erf (\chi _{2})+22\lambda ^{2} Q_{1}^{2}Q_{2}^{2}\beta ^{2}\sqrt{\pi }e^{\frac{\beta Q_{1}(\lambda ^2+Q_{2})^{2}}{\lambda ^{2}}}erf(\chi _{2})\\  &\quad -22\lambda ^{4} Q_{1}^{2}Q_{2}\beta ^{2}\sqrt{\pi }e^{\frac{\beta Q_{2}(\lambda ^2+Q_{2})^{2}}{\lambda ^{2}}}erf(\chi _{1}) \end{array}\right\} . \end{aligned}$$
- Vibrational entropy
  53 $$\begin{aligned} S(\beta )& {}=  k\ln Z(\beta ) - k\beta \frac{\partial \ln Z(\beta )}{\partial \beta } = k\ln e^{2\beta Q_{1}Q_{2}}\sqrt{\pi }\left[ \frac{\begin{array}{l} e^{2\beta Q_{1}Q_{2}}erf\left(\sqrt{-\beta Q_{1}}\lambda +\frac{Q_{2}\sqrt{-\beta Q_{1}}}{\lambda }\right)\\ \quad + e^{-2\beta Q_{1}Q_{2}}erf\left(\sqrt{-\beta Q_{1}}\lambda -\frac{Q_{2}\sqrt{-\beta Q_{1}}}{\lambda }\right)\end{array}}{4\sqrt{-\beta Q_{1}}}\right] \nonumber \\&\quad -\,\frac{1}{2}k\beta \left[ \frac{\begin{array}{l} 8Q_{1}Q_{2}\sqrt{\pi }\beta \lambda e^{2\beta Q_{1}Q_{2}}erf(\chi _{1}) + 2\lambda ^{2}\sqrt{-\beta Q_{1}}\chi _{3}+2Q_{2}\sqrt{-\beta Q_{1}}\chi _{3}\\ \quad + 2\lambda ^{2}\sqrt{-\beta Q_{1}}\chi _{4}-2Q_{2}\sqrt{-\beta Q_{1}}\chi _{4} - \sqrt{\pi }\lambda e^{2\beta Q_{1}Q_{2}}erf(\chi _{1}) \ \sqrt{\pi }\lambda e^{-2\beta Q_{1}Q_{2}}erf(\chi _{2})\end{array}}{\sqrt{\pi }\beta \lambda \left[ e^{2\beta Q_{1}Q_{2}}erf(\chi _{1})+e^{-2\beta Q_{1}Q_{2}}erf(\chi _{2})\right] }\right] \end{aligned}$$
- Vibrational free energy
  54 $$\begin{aligned} F(\beta )& {}=  -kT\ln Z(\beta ) = -\frac{1}{\beta }\ln e^{2\beta Q_{1}Q_{2}}\sqrt{\pi }\left[ \frac{\begin{array}{l} e^{2\beta Q_{1}Q_{2}}erf \left(\sqrt{-\beta Q_{1}}\lambda +\frac{Q_{2}\sqrt{-\beta Q_{1}}}{\lambda }\right)\\ \quad + e^{-2\beta Q_{1}Q_{2}}erf \left(\sqrt{-\beta Q_{1}}\lambda -\frac{Q_{2}\sqrt{-\beta Q_{1}}}{\lambda }\right)\end{array}}{4\sqrt{-\beta Q_{1}}}\right]. \end{aligned}$$

## Numerical results

Using Maple software package, the numerical solutions for spin and pseudospin symmetries were obtained using Eqs. (30) and (32) respectively. the nonrelativistic numerical bound state solutions were obtained using (35). Finally, the numerical solutions for Hulthen potential were obtained using (38) as one of the special cases in comparison to the results of existing literature.

## Discussion

**Figure 1:**
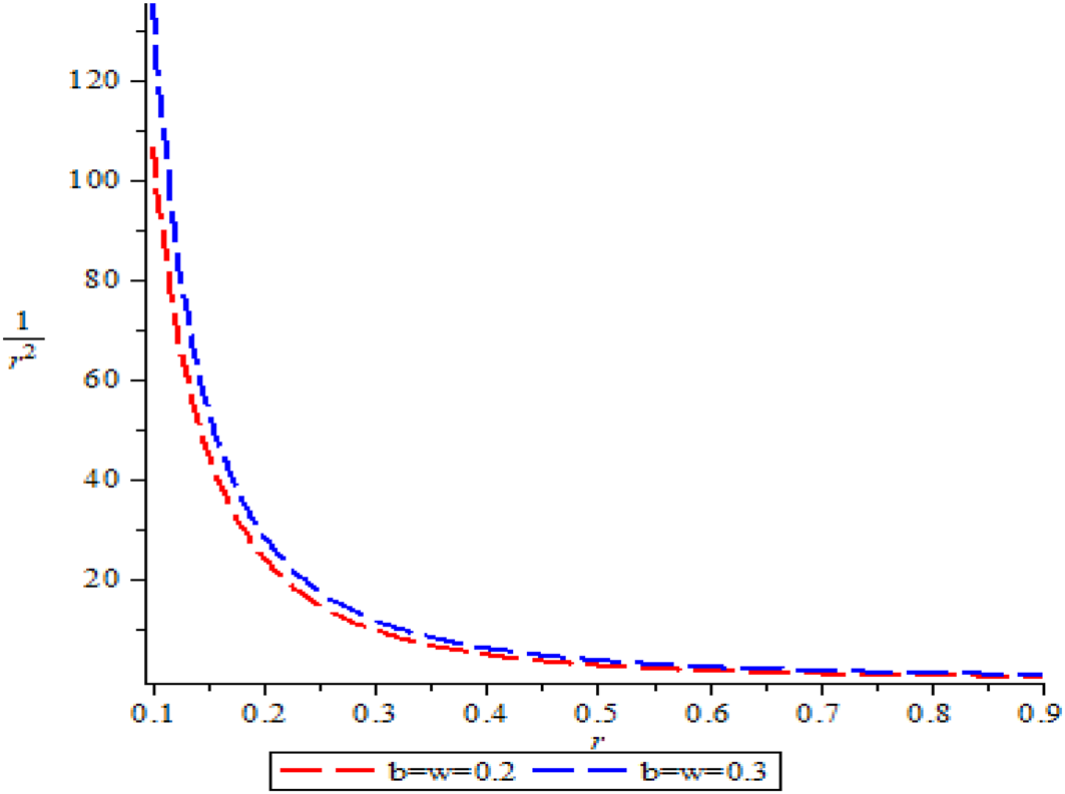
Standard Greene–Aldrich approximation to the centrifugal term.

**Figure 2:**
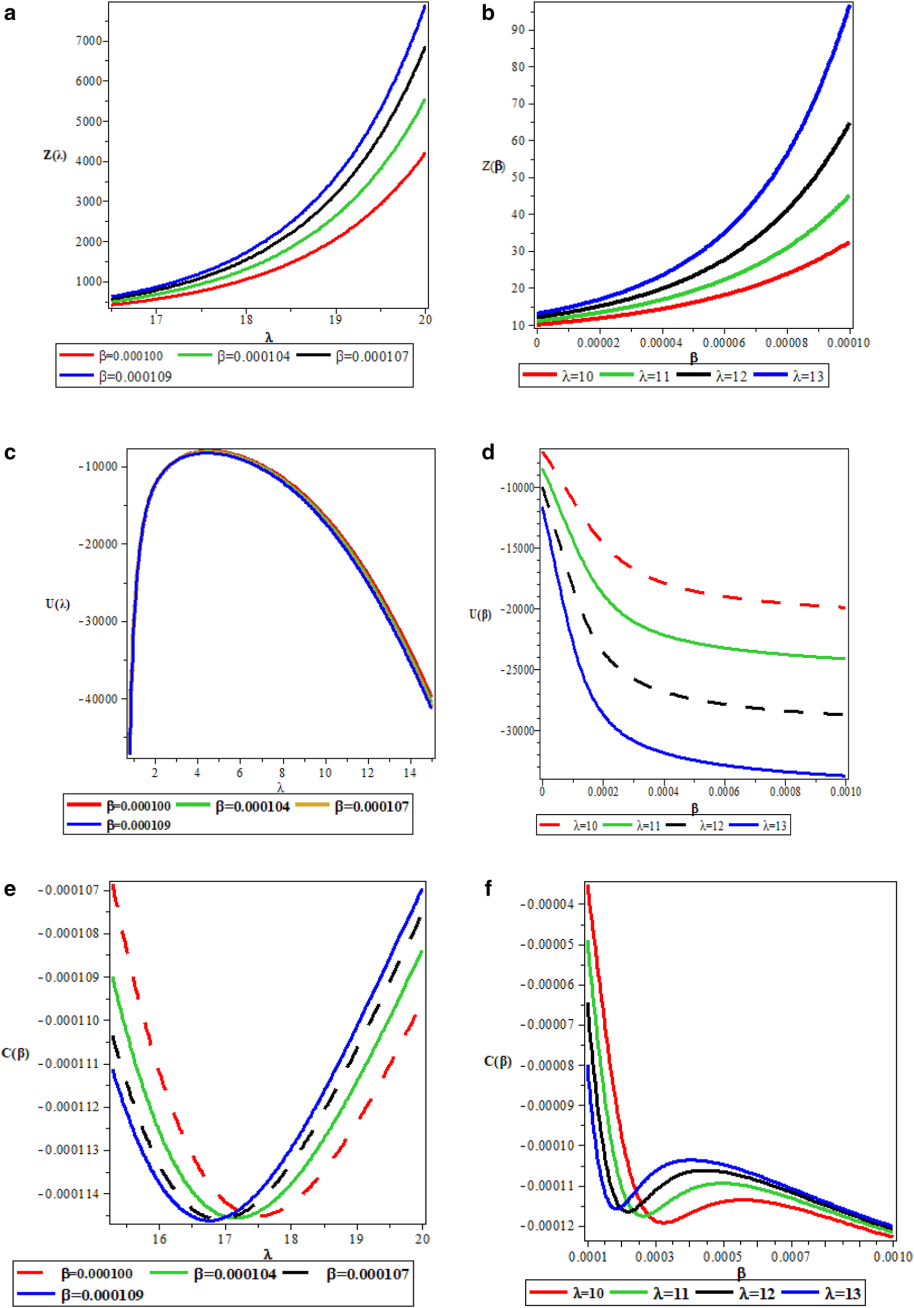

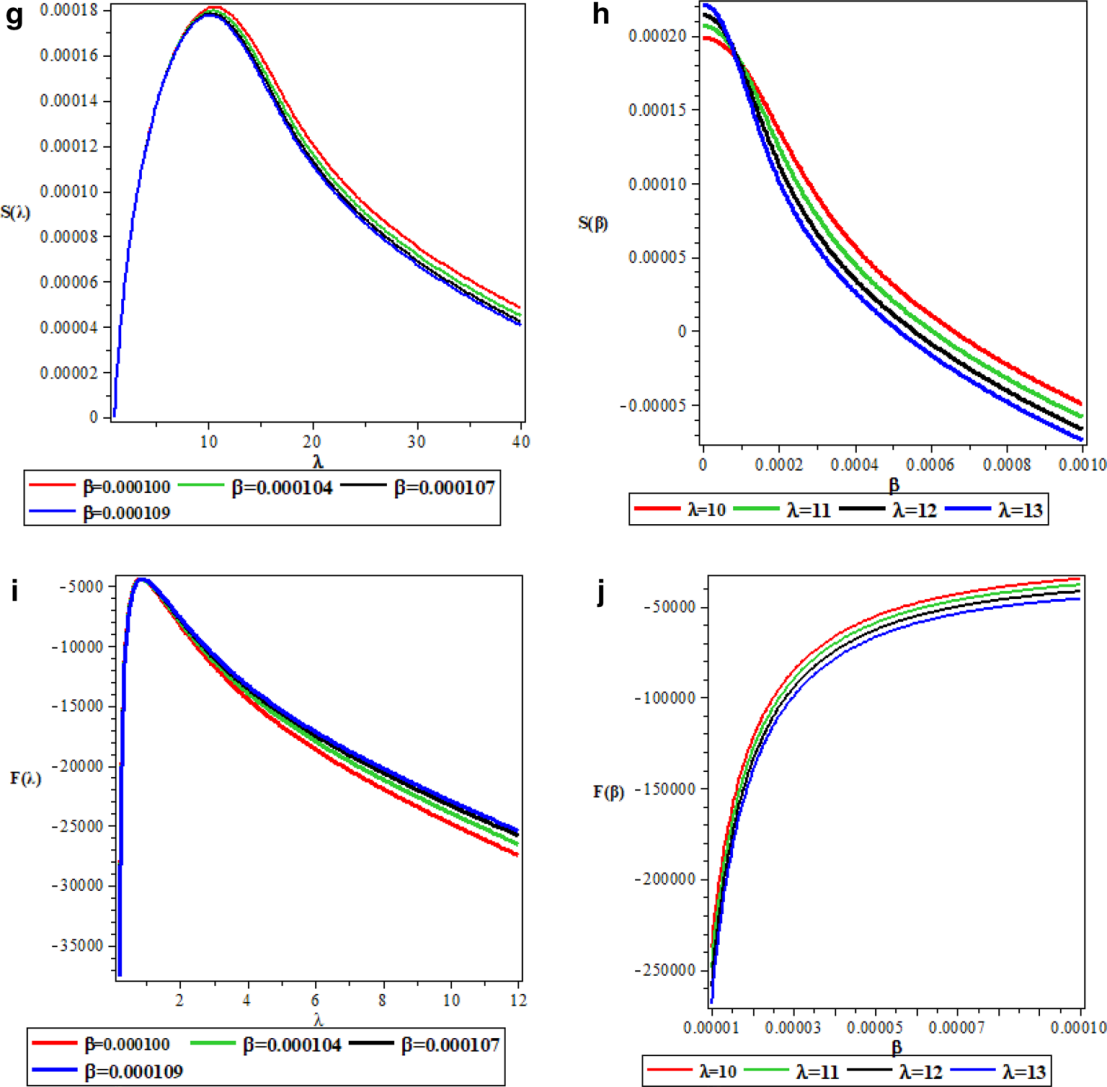
(**a**) Variation of partition function with respect to $\lambda$. (**b**) Variation of partition function with respect to $\beta$. (**c**) Variation of vibrational mean energy with respect to $\lambda$. (**d**) Variation of vibrational mean energy with respect to $\beta$. (**e**) Variation of specific heat capacity with respect to $\lambda$. (**f**) Variation of specific heat capacity with respect to $\beta$. (**g**) Variation of vibrational entropy with respect to $\lambda$. (**h**) Variation of vibrational entropy with respect to $\beta$. (**i**) Variation of vibrational free energy with respect to $\lambda$. (**j**) Variation of vibrational free energy with respect to $\beta$.

Figure 1 is the graph of standard Greene–Aldrich approximation to the centrifugal term. The trend of Fig. 1 shows that the approximation was suitable and appropriate for the proposed potential. Figure 2a,b are the partition function $Z(\lambda ,\beta )$ which increases exponentially with respect to $\lambda$ and $\beta$ respectively. The vibrational mean energy $U(\lambda , \beta )$ curves are shown in Fig. 2c,d. The vibrational mean energy decreases with temperature parameter $\beta$, but increases monotonically with a maximum turning point at $\lambda =4$ with respect to $\lambda$. The vibrational specific heat capacities $C(\lambda ,\beta )$ is shown in Fig. 2e,f, with $\lambda$; it has a perfect curve with a minimum turning point at different values of $\beta$. However, with $\beta$, it deceases and rises to a certain value and then decreases monotonically. The vibrational entropy $S(\lambda ,\beta )$ decreases with an increasing $\lambda$ and $\beta$ as shown in Fig. 2g,h respectively. The vibrational free energy $F(\lambda ,\beta )$ presented in Fig. 2i,j increases with increasing $\lambda$ and $\beta$ respectively. The behaviour of the thermal properties in the present study are in accordance with those reported in refs^68,71,72^. Table 1 is the numerical values for spin symmetry energies for $A=5fm^{-1}$, $V_{1}=15fm^{-1}$, $b=0.1$ and $M=10fm^{-1}$. Table 2 is the numerical values for spin symmetry energies for $A=5fm^{-1}$, $V_{1}=15fm^{-1}$, $b=0.2$ and $M=10fm^{-1}$. Table 3 is the numerical values for spin symmetry energies for $A=5fm^{-1}$, $V_{1}=15fm^{-1}$, $b=0.3$ and $M=10fm^{-1}$ while Table 4 is the numerical values for spin symmetry energies for $A=5fm^{-1}$, $V_{1}=15fm^{-1}$, $b=0.3$ and $M=10fm^{-1}$. In Table 2, the S-spin quantum state $0s_{\frac{1}{2}}$ has the highest relativistic spin energy symmetry value of 10.65566240$fm^{-1}$ for $\kappa <0$ and the minimum value of − 9.402539935$fm^{-1}$ for $\kappa >0$. In Table 3, the S-spin quantum state $0s_{\frac{1}{2}}$ has the highest relativistic spin energy symmetry value of 11.94562892$fm^{-1}$ for $\kappa <0$ and the minimum value of − 9.653034948$fm^{-1}$ for $\kappa >0$. In Table 4, the S-spin quantum state $0s_{\frac{1}{2}}$ has the highest relativistic spin energy symmetry value of 13.56961400$fm^{-1}$ for $\kappa <0$ and the minimum value of − 9.759490473$fm^{-1}$ for $\kappa >0$. This illustrates that the s-spin quantum state assume the most stable state for relativistic spin energy symmetry. Also, Tables 1, 2, 3 and 4 are predominantly positive values which are applicable in describing some properties of relativistic antiparticles. Considering Tables 1, 2, 3 and4, the energy rise with an increase in the screening parameter. The following degeneracies were obtain for the screening parameter $b=0.1, 0.2, 0.3$ and 0.4: $3p_{\frac{3}{2}}=3s_{\frac{1}{2}}$, $2f_{\frac{7}{2}}=2d_{\frac{5}{2}}$, $3f_{\frac{7}{2}}=3d_{\frac{5}{2}}$, $0f_{\frac{7}{2}}=0d_{\frac{5}{2}}$ and $1f_{\frac{7}{2}}=1d_{\frac{5}{2}}$.

**Table 1:** Numerical values for spin symmetry energies for $A=5fm^{-1}, v_{1}=15fm^{-1}, b=0.1, M=10fm^{-1}, (\hbar =c=1)\text {unit}$.

$\ell$	$k<0$	*n*	$(\ell ,j)$	$E_{nk}$	$k>0$	*n*	$(\ell ,j)$	$E_{nk}$
0	− 1	0	0$s_{\frac{1}{2}}$	8.983921278	1	0	0$s_{\frac{1}{2}}$	− 8.223233399
0	− 1	1	1$s_{\frac{1}{2}}$	3.255885745	1	1	1$s_{\frac{1}{2}}$	2.349810532
0	− 1	2	2$s_{\frac{1}{2}}$	3.764133532	1	2	2$s_{\frac{1}{2}}$	3.244054374
0	− 1	3	3$s_{\frac{1}{2}}$	4.671917081	1	3	3$s_{\frac{1}{2}}$	4.275589604
1	− 2	0	0$p_{\frac{3}{2}}$	− 8.223233399	2	0	0$p_{\frac{3}{2}}$	− 7.512110433
1	− 2	1	1$p_{\frac{3}{2}}$	2.349810532	2	1	1$p_{\frac{3}{2}}$	0.6388962715
1	− 2	2	2$p_{\frac{3}{2}}$	3.244054374	2	2	2$p_{\frac{3}{2}}$	2.246255163
1	− 2	3	3$p_{\frac{3}{2}}$	4.275589604	2	3	3$p_{\frac{3}{2}}$	3.505707617
2	− 3	0	0$d_{\frac{5}{2}}$	− 7.512110433	3	0	0$d_{\frac{5}{2}}$	− 7.240908451
2	− 3	1	1$d_{\frac{5}{2}}$	0.6388962715	3	1	1$d_{\frac{5}{2}}$	− 1.407169997
2	− 3	2	2$d_{\frac{5}{2}}$	2.246255163	3	2	2$d_{\frac{5}{2}}$	0.8693835435
2	− 3	3	3$d_{\frac{5}{2}}$	3.505707617	3	3	3$d_{\frac{5}{2}}$	2.410060646
3	− 4	0	0$f_{\frac{7}{2}}$	− 7.240908451	4	0	0$f_{\frac{7}{2}}$	− 7.586751966
3	− 4	1	1$f_{\frac{7}{2}}$	− 1.407169997	4	1	1$f_{\frac{7}{2}}$	− 3.30875422
3	− 4	2	2$f_{\frac{7}{2}}$	0.8693835435	4	2	2$f_{\frac{7}{2}}$	− 0.7385660036
3	− 4	3	3$f_{\frac{7}{2}}$	2.410060646	4	3	3$f_{\frac{7}{2}}$	1.060283723

**Table 2:** Numerical values for spin symmetry energies for $A=5fm^{-1}, v_{1}=15fm^{-1}, b=0.2, M=10fm^{-1}, (\hbar =c=1)\text {unit}$.

$\ell$	$k<0$	*n*	$(\ell ,j)$	$E_{nk}$	$k>0$	*n*	$(\ell ,j)$	$E_{nk}$
0	− 1	0	0$s_{\frac{1}{2}}$	10.65566240	1	0	0$s_{\frac{1}{2}}$	− 9.402539935
0	− 1	1	1$s_{\frac{1}{2}}$	8.879147410	1	1	1$s_{\frac{1}{2}}$	8.593038708
0	− 1	2	2$s_{\frac{1}{2}}$	6.851894743	1	2	2$s_{\frac{1}{2}}$	6.412449813
0	− 1	3	3$s_{\frac{1}{2}}$	6.591830427	1	3	3$s_{\frac{1}{2}}$	6.277759731
1	− 2	0	0$p_{\frac{3}{2}}$	− 9.402539935	2	0	0$p_{\frac{3}{2}}$	− 8.862687863
1	− 2	1	1$p_{\frac{3}{2}}$	8.593038708	2	1	1$p_{\frac{3}{2}}$	8.010408756
1	− 2	2	2$p_{\frac{3}{2}}$	6.412449813	2	2	2$p_{\frac{3}{2}}$	5.467061490
1	− 2	3	3$p_{\frac{3}{2}}$	6.257759731	2	3	3$p_{\frac{3}{2}}$	5.589744715
2	− 3	0	0$d_{\frac{5}{2}}$	− 8.862687863	3	0	0$d_{\frac{5}{2}}$	− 8.201366952
2	− 3	1	1$d_{\frac{5}{2}}$	8.010408756	3	1	1$d_{\frac{5}{2}}$	− 5.105451949
2	− 3	2	2$d_{\frac{5}{2}}$	5.467061490	3	2	2$d_{\frac{5}{2}}$	3.898663797
2	− 3	3	3$d_{\frac{5}{2}}$	5.589744715	3	3	3$d_{\frac{5}{2}}$	4.606019730
3	− 4	0	0$f_{\frac{7}{2}}$	− 8.201366952	4	0	0$f_{\frac{7}{2}}$	− 7.510682389
3	− 4	1	1$f_{\frac{7}{2}}$	− 5.105451949	4	1	1$f_{\frac{7}{2}}$	− 3.748571939
3	− 4	2	2$f_{\frac{7}{2}}$	3.898663797	4	2	2$f_{\frac{7}{2}}$	1.865629960
3	− 4	3	3$f_{\frac{7}{2}}$	4.606019730	4	3	3$f_{\frac{7}{2}}$	3.379690499

**Table 3:** Numerical values for spin symmetry energies for $A=5fm^{-1}, v_{1}=15fm^{-1}, b=0.3, M=10fm^{-1}, (\hbar =c=1)\text {unit}$.

$\ell$	$k<0$	*n*	$(\ell ,j)$	$E_{nk}$	$k>0$	*n*	$(\ell ,j)$	$E_{nk}$
0	− 1	0	0$s_{\frac{1}{2}}$	11.94562892	1	0	0$s_{\frac{1}{2}}$	− 9.653054948
0	− 1	1	1$s_{\frac{1}{2}}$	11.45354051	1	1	1$s_{\frac{1}{2}}$	11.30816398
0	− 1	2	2$s_{\frac{1}{2}}$	10.64354064	1	2	2$s_{\frac{1}{2}}$	10.45259601
0	− 1	3	3$s_{\frac{1}{2}}$	9.931274480	1	3	3$s_{\frac{1}{2}}$	9.710639231
1	− 2	0	0$p_{\frac{3}{2}}$	− 9.653054948	2	0	0$p_{\frac{3}{2}}$	− 9.226504128
1	− 2	1	1$p_{\frac{3}{2}}$	11.30816398	2	1	1$p_{\frac{3}{2}}$	11.01670360
1	− 2	2	2$p_{\frac{3}{2}}$	10.45259601	2	2	2$p_{\frac{3}{2}}$	10.05799030
1	− 2	3	3$p_{\frac{3}{2}}$	9.710639231	2	3	3$p_{\frac{3}{2}}$	9.251447327
2	− 3	0	0$d_{\frac{5}{2}}$	− 9.226504128	3	0	0$d_{\frac{5}{2}}$	− 8.642597290
2	− 3	1	1$d_{\frac{5}{2}}$	11.01670360	3	1	1$d_{\frac{5}{2}}$	− 7.704698788
2	− 3	2	2$d_{\frac{5}{2}}$	10.05799030	3	2	2$d_{\frac{5}{2}}$	9.428222506
2	− 3	3	3$d_{\frac{5}{2}}$	9.251447327	3	3	3$d_{\frac{5}{2}}$	8.510496320
3	− 4	0	0$f_{\frac{7}{2}}$	− 8.642597290	4	0	0$f_{\frac{7}{2}}$	− 7.911699446
3	− 4	1	1$f_{\frac{7}{2}}$	− 7.704698788	4	1	1$f_{\frac{7}{2}}$	− 6.620201873
3	− 4	2	2$f_{\frac{7}{2}}$	9.428222506	4	2	2$f_{\frac{7}{2}}$	8.493229223
3	− 4	3	3$f_{\frac{7}{2}}$	8.510496320	4	3	3$f_{\frac{7}{2}}$	7.400643640

**Table 4:** Numerical values for spin symmetry energies for $A=5fm^{-1}, v_{1}=15fm^{-1}, b=0.4, M=10fm^{-1}, (\hbar =c=1)\text {unit}$.

$\ell$	$k<0$	*n*	$(\ell ,j)$	$E_{nk}$	$k>0$	*n*	$(\ell ,j)$	$E_{nk}$
0	− 1	0	0$s_{\frac{1}{2}}$	13.56961400	1	0	0$s_{\frac{1}{2}}$	− 9.759490473
0	− 1	1	1$s_{\frac{1}{2}}$	13.42954857	1	1	1$s_{\frac{1}{2}}$	13.32679497
0	− 1	2	2$s_{\frac{1}{2}}$	13.18355639	1	2	2$s_{\frac{1}{2}}$	13.7149132
0	− 1	3	3$s_{\frac{1}{2}}$	12.89651062	1	3	3$s_{\frac{1}{2}}$	12.77434725
1	− 2	0	0$p_{\frac{3}{2}}$	− 9.759490473	2	0	0$p_{\frac{3}{2}}$	− 9.405058745
1	− 2	1	1$p_{\frac{3}{2}}$	13.32679497	2	1	1$p_{\frac{3}{2}}$	− 9.212559444
1	− 2	2	2$p_{\frac{3}{2}}$	13.07149132	2	2	2$p_{\frac{3}{2}}$	12.84444065
1	− 2	3	3$p_{\frac{3}{2}}$	12.77434725	2	3	3$p_{\frac{3}{2}}$	12.52547716
2	− 3	0	0$d_{\frac{5}{2}}$	− 9.405058745	3	0	0$d_{\frac{5}{2}}$	− 8.898795585
2	− 3	1	1$d_{\frac{5}{2}}$	− 9.212559444	3	1	1$d_{\frac{5}{2}}$	− 8.562364622
2	− 3	2	2$d_{\frac{5}{2}}$	12.8444065	3	2	2$d_{\frac{5}{2}}$	12.49608256
2	− 3	3	3$d_{\frac{5}{2}}$	12.52547716	3	3	3$d_{\frac{5}{2}}$	12.13969691
3	− 4	0	0$f_{\frac{7}{2}}$	− 8.898795585	4	0	0$f_{\frac{7}{2}}$	− 8.238455058
3	− 4	1	1$f_{\frac{7}{2}}$	− 8.562364622	4	1	1$f_{\frac{7}{2}}$	− 7.749637734
3	− 4	2	2$f_{\frac{7}{2}}$	12.49608256	4	2	2$f_{\frac{7}{2}}$	− 7.377835097
3	− 4	3	3$f_{\frac{7}{2}}$	12.1396969	4	3	3$f_{\frac{7}{2}}$	11.59818143

**Figure 3:**
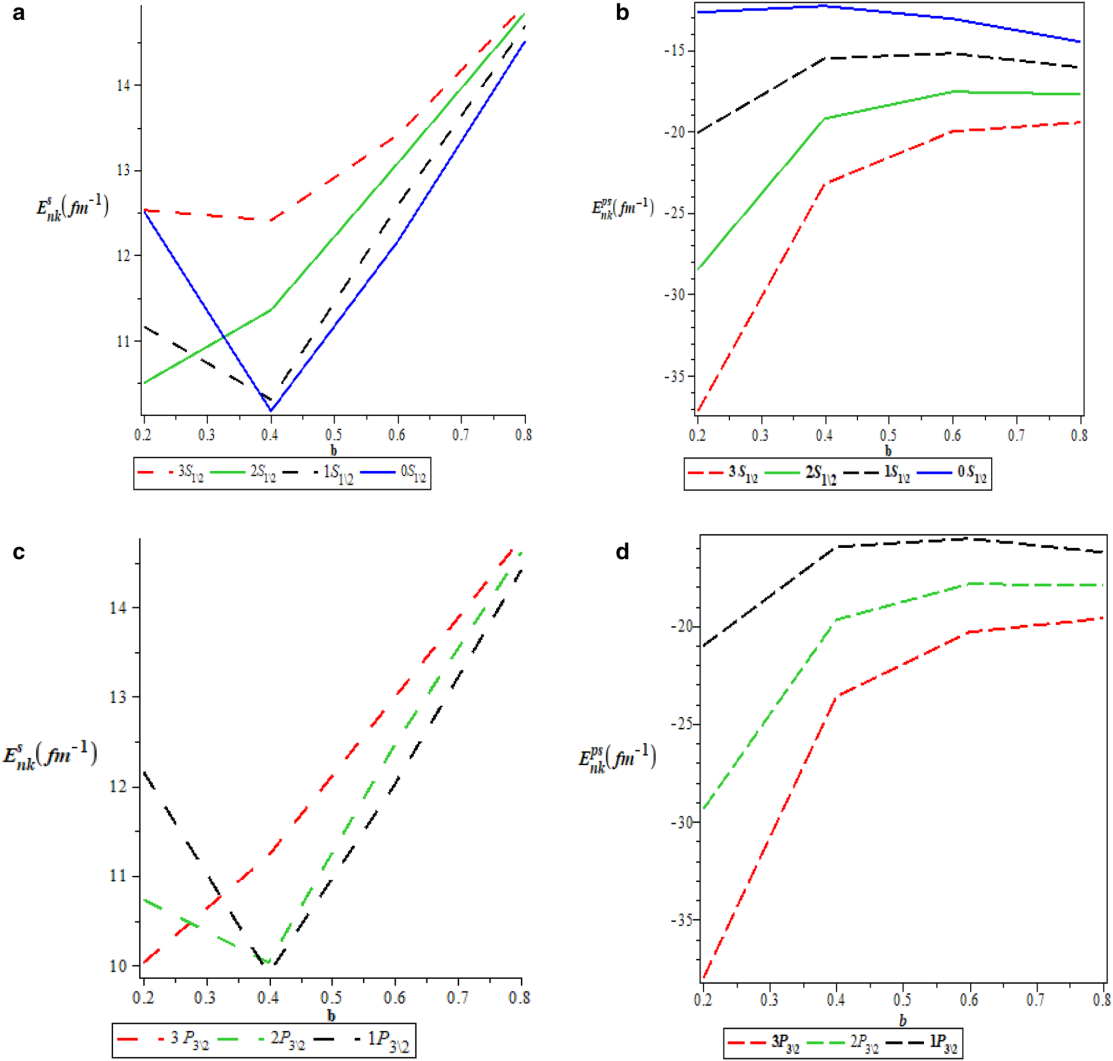
(**a**) Variation of Energy Spectra for S-spin Symmetry. (**b**) Variation of Energy Spectra for S-pseudospin Symmetry. (**c**) Variation of Energy Spectra for P-spin Symmetry. (**d**) Variation of Energy Spectra for P-pseudospin Symmetry.

**Figure 4:**
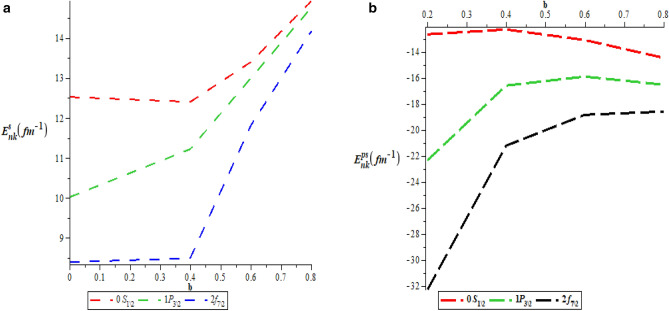
(**a**) Variation of Energy Spectra for Mixed Quantum spin. (**b**) Variation of Energy Spectra for Mixed Pseudo Quantum spin.

**Figure 5:**
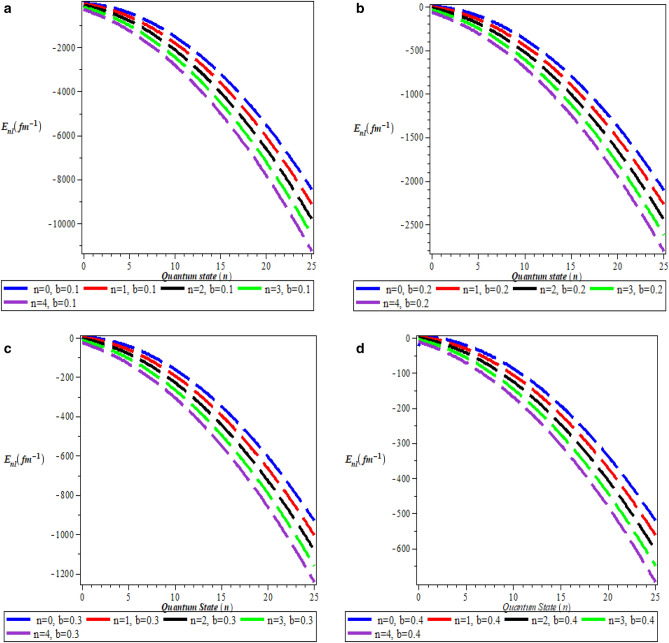
(**a**) Nonrelativistic energy spectra for $\hbox {b} = 0.1$. (**b**) Nonrelativistic energy spectra for $\hbox {b} = 0.2$. (**c**) Nonrelativistic energy spectra for $\hbox {b} = 0.3$. (**d**) Nonrelativistic energy spectra for $\hbox {b} = 0.4$.

Table 5 is the numerical values for pseudospin symmetry for $A=5fm^{-1}$, $V_{1}=15fm^{-1}$, $b=0.1$ and $M=10fm^{-1}$. Table 6 is the numerical values for pseudospin symmetry for $A=5fm^{-1}$, $V_{1}=15fm^{-1}$, $b=0.2$ and $M=10fm^{-1}$, Table 7 is the numerical values for pseudospin symmetry for $A=5fm^{-1}$, $V_{1}=15fm^{-1}$, $b=0.3$ and $M=10fm^{-1}$ and Table 8 is the numerical values for pseudospin symmetry for $A=5fm^{-1}$, $V_{1}=15fm^{-1}$, $b=0.4$ and $M=10fm^{-1}$. Tables 5, 6, 7 and 8 are the numerical relativistic pseudospin bound state energies which constitutes negative energy eigenvalues which can be use to describe the properties of relativistic particles. The degeneracies obtained for the pseudospin limit with the same screening parameter are: $3s_{\frac{1}{2}}=3p_{\frac{3}{2}}$, $0p_{\frac{3}{2}}=0d_{\frac{5}{2}}$, $2s_{\frac{1}{2}}=2p_{\frac{3}{2}}$, $3p_{\frac{3}{2}}=3d_{\frac{5}{2}}$,$2d_{\frac{5}{2}}=2f_{\frac{7}{2}}$, $1d_{\frac{5}{2}}=1f_{\frac{7}{2}}$. Figure 3a is the variation of energy spectra for S-quantum spin symmetry. Figure 3b is the variation of energy spectra for s-quantum pseudospin symmetry. Figure 3c is the variation of energy spectra for p-quantum spin symmetry while Fig. 3d is the variation of energy spectra for P-quantum pseudospin symmetry. Considering Fig. 3a–d, there is an inverse relationship between the spin and pseudospin energy spectra. spin spectral diagram is an exact opposite of pseudospin diagram. Figure 4a is the variation of energy spectra for a mixed quantum spin state while Fig. 4b is the variation of energy spectra for a mixed pseudo quantum spin. There is also an inverse relationship between the mixed spin quantum state and the mixed pseudo quantum spin as observed in Fig. 3a–d Meanwhile, the numerical bound state energies decreases with an increase in quantum state for both spin and pseudospin symmetries. Table 9 is nonrelativistic numerical bound state energies for different quantum state obtain using Eq. (35). The energy eigenvalues of Table 9 are predominantly negative which is the necessary and sufficient condition for bound state. Figure 5a is the nonrelativistic energy spectra for the screening parameter $b=0.1$, Fig. 5b is the nonrelativistic energy spectra for the screening parameter $b=0.2$, Fig. 5c is the nonrelativistic energy spectra for the screening parameter $b=0.3$ and Fig. 5d is the nonrelativistic energy spectra for the screening parameter $b=0.4$. Figure 5a–d show unique quantisation of different energy level which is very important concept in quantum physics in the description of atomic structure.

**Table 5:** Numerical values for pseudospin symmetry energies for $A=5fm^{-1}, v_{1}=15fm^{-1}, b=0.1, M=10fm^{-1}, (\hbar =c=1)\text {unit}$.

$\ell$	$k<0$	*n*	$(\ell ,j)$	$E_{nk}$	$k>0$	*n*	$(\ell ,j)$	$E_{nk}$
0	− 1	0	0$s_{\frac{1}{2}}$	− 12.69213560	1	0	0$s_{\frac{1}{2}}$	− 12.30271714
0	− 1	1	1$s_{\frac{1}{2}}$	− 20.05606687	1	1	1$s_{\frac{1}{2}}$	− 19.58322167
0	− 1	2	2$s_{\frac{1}{2}}$	− 28.51380132	1	2	2$s_{\frac{1}{2}}$	− 28.07267520
0	− 1	3	3$s_{\frac{1}{2}}$	− 37.19205174	1	3	3$s_{\frac{1}{2}}$	− 36.79032895
1	− 2	0	0$p_{\frac{3}{2}}$	− 13.50050752	2	0	0$p_{\frac{3}{2}}$	− 12.69213560
1	− 2	1	1$p_{\frac{3}{2}}$	− 20.98411765	2	1	1$p_{\frac{3}{2}}$	− 20.05606687
1	− 2	2	2$p_{\frac{3}{2}}$	− 29.37941053	2	2	2$p_{\frac{3}{2}}$	− 28.51380132
1	− 2	3	3$p_{\frac{3}{2}}$	− 37.98303228	2	3	3$p_{\frac{3}{2}}$	− 37.19205174
2	− 3	0	0$d_{\frac{5}{2}}$	− 14.75172391	3	0	0$d_{\frac{5}{2}}$	− 13.50050752
2	− 3	1	1$d_{\frac{5}{2}}$	− 22.33384979	3	1	1$d_{\frac{5}{2}}$	− 20.98411765
2	− 3	2	2$d_{\frac{5}{2}}$	− 30.63932420	3	2	2$d_{\frac{5}{2}}$	− 29.37941053
2	− 3	3	3$d_{\frac{5}{2}}$	− 39.14034408	3	3	3$d_{\frac{5}{2}}$	− 37.98303228
3	− 4	0	0$f_{\frac{7}{2}}$	− 16.43288886	4	0	0$f_{\frac{7}{2}}$	− 14.75172391
3	− 4	1	1$f_{\frac{7}{2}}$	− 24.06031886	4	1	1$f_{\frac{7}{2}}$	− 22.33384979
3	− 4	2	2$f_{\frac{7}{2}}$	− 32.25483876	4	2	2$f_{\frac{7}{2}}$	− 30.63932420
3	− 4	3	3$f_{\frac{7}{2}}$	− 40.63390244	4	3	3$f_{\frac{7}{2}}$	− 39.14034408

**Table 6:** Numerical values for pseudospin symmetry energies for $A=5fm^{-1}, v_{1}=15fm^{-1}, b=0.2, M=10fm^{-1}, (\hbar =c=1)\text {unit}$.

$\ell$	$k<0$	*n*	$(\ell ,j)$	$E_{nk}$	$k>0$	*n*	$(\ell ,j)$	$E_{nk}$
0	− 1	0	0$s_{\frac{1}{2}}$	− 12.28432355	1	0	0$s_{\frac{1}{2}}$	− 12.09991279
0	− 1	1	1$s_{\frac{1}{2}}$	− 15.51562769	1	1	1$s_{\frac{1}{2}}$	− 15.29325472
0	− 1	2	2$s_{\frac{1}{2}}$	− 19.25297714	1	2	2$s_{\frac{1}{2}}$	− 19.02772195
0	− 1	3	3$s_{\frac{1}{2}}$	− 23.19071008	1	3	3$s_{\frac{1}{2}}$	− 22.97175688
1	− 2	0	0$p_{\frac{3}{2}}$	− 12.66108726	2	0	0$p_{\frac{3}{2}}$	− 12.28432355
1	− 2	1	1$p_{\frac{3}{2}}$	− 15.95750279	2	1	1$p_{\frac{3}{2}}$	− 15.51562769
1	− 2	2	2$p_{\frac{3}{2}}$	− 19.70093833	2	2	2$p_{\frac{3}{2}}$	− 19.02772195
1	− 2	3	3$p_{\frac{3}{2}}$	− 23.62302141	2	3	3$p_{\frac{3}{2}}$	− 23.19071008
2	− 3	0	0$d_{\frac{5}{2}}$	− 13.24035926	3	0	0$d_{\frac{5}{2}}$	− 12.66108726
2	− 3	1	1$d_{\frac{5}{2}}$	− 16.61218365	3	1	1$d_{\frac{5}{2}}$	− 15.95750279
2	− 3	2	2$d_{\frac{5}{2}}$	− 20.35795593	3	2	2$d_{\frac{5}{2}}$	− 19.70093833
2	− 3	3	3$d_{\frac{5}{2}}$	− 24.25815897	3	3	3$d_{\frac{5}{2}}$	− 23.62302141
3	− 4	0	0$f_{\frac{7}{2}}$	− 14.02686986	4	0	0$f_{\frac{7}{2}}$	− 16.61218365
3	− 4	1	1$f_{\frac{7}{2}}$	− 17.46826708	4	1	1$f_{\frac{7}{2}}$	− 16.61218365
3	− 4	2	2$f_{\frac{7}{2}}$	− 21.21020722	4	2	2$f_{\frac{7}{2}}$	− 20.35795593
3	− 4	3	3$f_{\frac{7}{2}}$	− 25.08181569	4	3	3$f_{\frac{7}{2}}$	− 24.25815897

**Table 7:** Numerical values for pseudospin symmetry energies for $A=5fm^{-1}, v_{1}=15fm^{-1}, b=0.3, M=10fm^{-1}, (\hbar =c=1)\text {unit}$.

$\ell$	$k<0$	*n*	$(\ell ,j)$	$E_{nk}$	$k>0$	*n*	$(\ell ,j)$	$E_{nk}$
0	− 1	0	0$s_{\frac{1}{2}}$	− 13.10449580	1	0	0$s_{\frac{1}{2}}$	− 12.97416326
0	− 1	1	1$s_{\frac{1}{2}}$	− 15.24478036	1	1	1$s_{\frac{1}{2}}$	− 15.10293877
0	− 1	2	2$s_{\frac{1}{2}}$	− 17.57946118	1	2	2$s_{\frac{1}{2}}$	− 17.43537205
0	− 1	3	3$s_{\frac{1}{2}}$	− 20.01181246	1	3	3$s_{\frac{1}{2}}$	− 19.86975971
1	− 2	0	0$p_{\frac{3}{2}}$	− 13.365886250	2	0	0$p_{\frac{3}{2}}$	− 13.10449580
1	− 2	1	1$p_{\frac{3}{2}}$	− 15.52663385	2	1	1$p_{\frac{3}{2}}$	− 15.24478036
1	− 2	2	2$p_{\frac{3}{2}}$	− 17.86488937	2	2	2$p_{\frac{3}{2}}$	− 17.57946118
1	− 2	3	3$p_{\frac{3}{2}}$	− 20.29298159	2	3	3$p_{\frac{3}{2}}$	− 20.01181246
2	− 3	0	0$d_{\frac{5}{2}}$	− 13.75860256	3	0	0$d_{\frac{5}{2}}$	− 17.57946118
2	− 3	1	1$d_{\frac{5}{2}}$	− 15.94464389	3	1	1$d_{\frac{5}{2}}$	− 1.552663385
2	− 3	2	2$d_{\frac{5}{2}}$	− 18.28631249	3	2	2$d_{\frac{5}{2}}$	− 17.86488937
2	− 3	3	3$d_{\frac{5}{2}}$	− 20.70765586	3	3	3$d_{\frac{5}{2}}$	− 20.29298159
3	− 4	0	0$f_{\frac{7}{2}}$	− 14.28119674	4	0	0$f_{\frac{7}{2}}$	− 13.75860256
3	− 4	1	1$f_{\frac{7}{2}}$	− 16.49274719	4	1	1$f_{\frac{7}{2}}$	− 15.94464389
3	− 4	2	2$f_{\frac{7}{2}}$	− 18.83612438	4	2	2$f_{\frac{7}{2}}$	− 18.28631249
3	− 4	3	3$f_{\frac{7}{2}}$	− 21.24802969	4	3	3$f_{\frac{7}{2}}$	− 20.70765586

**Table 8:** Numerical values for pseudospin symmetry energies for $A=5fm^{-1}, v_{1}=15fm^{-1}, b=0.4, M=10fm^{-1}, (\hbar =c=1)\text {unit}$.

$\ell$	$k<0$	*n*	$(\ell ,j)$	$E_{nk}$	$k>0$	*n*	$(\ell ,j)$	$E_{nk}$
0	− 1	0	0$s_{\frac{1}{2}}$	− 14.47035819	1	0	0$s_{\frac{1}{2}}$	− 14.37133775
0	− 1	1	1$s_{\frac{1}{2}}$	− 16.06990567	1	1	1$s_{\frac{1}{2}}$	− 15.96860262
0	− 1	2	2$s_{\frac{1}{2}}$	− 17.735611113	1	2	2$s_{\frac{1}{2}}$	− 17.63433738
0	− 1	3	3$s_{\frac{1}{2}}$	− 19.43972082	1	3	3$s_{\frac{1}{2}}$	− 19.33973124
1	− 2	0	0$p_{\frac{3}{2}}$	− 14.66732944	2	0	0$p_{\frac{3}{2}}$	− 14.47035819
1	− 2	1	1$p_{\frac{3}{2}}$	− 16.27093685	2	1	1$p_{\frac{3}{2}}$	− 16.06990567
1	− 2	2	2$p_{\frac{3}{2}}$	− 17.93639247	2	2	2$p_{\frac{3}{2}}$	− 17.73561113
1	− 2	3	3$p_{\frac{3}{2}}$	− 19.63790665	2	3	3$p_{\frac{3}{2}}$	− 19.43972082
2	− 3	0	0$d_{\frac{5}{2}}$	− 14.96002329	3	0	0$d_{\frac{5}{2}}$	− 14.66732944
2	− 3	1	1$d_{\frac{5}{2}}$	− 16.56860653	3	1	1$d_{\frac{5}{2}}$	− 16.27093685
2	− 3	2	2$d_{\frac{5}{2}}$	− 18.23327378	3	2	2$d_{\frac{5}{2}}$	− 17.93639247
2	− 3	3	3$d_{\frac{5}{2}}$	− 19.93084754	3	3	3$d_{\frac{5}{2}}$	− 19.63790665
3	− 4	0	0$f_{\frac{7}{2}}$	− 15.34495615	4	0	0$f_{\frac{7}{2}}$	− 14.96002329
3	− 4	1	1$f_{\frac{7}{2}}$	− 16.95844684	4	1	1$f_{\frac{7}{2}}$	− 16.56860653
3	− 4	2	2$f_{\frac{7}{2}}$	− 18.62144186	4	2	2$f_{\frac{7}{2}}$	− 18.23327378
3	− 4	3	3$f_{\frac{7}{2}}$	− 20.31372099	4	3	3$f_{\frac{7}{2}}$	− 19.93084754

**Table 9:** Nonrelativistic numerical solutions for $A=5fm^{-1}, v_{1}=15fm^{-1}, b=0.1, (\hbar =c=1)\text {unit}$.

*n*	*l*	$E_{nl},\,b = 0.1$	$E_{nl},\,b = 0.2$	$E_{nl},\,b = 0.3$	$E_{nl},\,b = 0.4$
0	0	− 12.0181250	− 2.3225000	0.520138889	1.30375000
1	0	− 87.4934896	− 18.9583333	− 6.00622106	− 1.16536458
	1	− 109.063125	− 24.5025000	− 8.56812500	− 2.66625000
2	0	− 182.670166	− 40.7539062	− 14.4047580	− 5.10180664
	1	− 214.749043	− 48.4604592	− 17.6280116	− 6.79145408
	2	− 303.153125	− 68.8625000	− 25.6003472	− 10.606250
3	0	− 279.82502	− 64.0625000	− 24.1323031	− 10.1872417
	0	− 311.98140	− 71.7850000	− 27.3604340	− 11.8782812
	0	− 602.045000	− 89.597656	− 34.5408257	− 15.4670410
	0	− 279.82502	− 142.805000	− 57.9605556	− 28.5012500
4	0	− 387.471458	− 90.6358333	− 35.7246065	− 16.5745833
	1	− 417.501993	− 97.9209184	− 38.8189573	− 18.2274394
	2	− 482.44108	− 113.49479	− 45.3146340	− 21.6207682
	3	− 725.170139	− 174.222222	− 72.3337191	− 36.8368056
	3	− 1003.75451	− 244.436412	− 103.906246	− 54.8202637
5	0	− 513.819102	− 122.163125	− 49.6983030	− 24.4112891
	1	− 542.076047	− 129.083276	− 52.6810413	− 26.0322888
	2	− 600.711343	− 143.360078	− 58.7800185	− 29.3123919
	3	− 879.002551	− 213.063776	− 89.8438209	− 46.8373724
	4	− 1204.49792	− 294.812302	− 126.418037	− 67.5580482
	5	− 12.0181250	− 369.920000	− 159.906806	− 86.4612500

Finally, using Eq. (38) the numerical bound state solution is obtain for Hulthen potential as shown in Table 10. The numerical solutions obtained for Hulthen potential are in excellent agreement with Refs^73–77^.

**Table 10:** Comparison of energy spectra $(-E_{nl})$ for Hulthen potential as a function of the screening parameter $\frac{1}{b}$ for 2*p*, 3*p*,3*d*, 4*p*, 4*d*, 4*f*, 5*p* $(\hbar =c=1)\,\,\text {unit}$.

State	1/*b*	Present	Jia et al.^73^	Ikhdair^74^	Bayrak et al.^75^	Varshni^76^	Stanek^77^
2*p*	0.025	0.1128125	0.1126344	0.1127611	0.1127605	0.1127605	0.1127604
	0.050	0.1012500	0.1009128	0.1010442	0.1010425	0.1010425	0.101042
	0.075	0.0903120	0.0898350	0.0898495	0.0898478	0.0898478	0.0898453
	0.100	0.0800000	0.0794011	0.0791769	0.0791794	0.0791794	0.0791717
	0.150	0.0612500	0.0604650	0.0593981	0.0594415	0.0594415	0.0594007
	0.200	0.0450000	0.0441045	0.0417078	0.0418861	0.0418860	0.0417491
	0.250	0.0312500	0.0303195	0.0261059	0.0266111	0.0266111	0.0262466
	0.300	0.0199994	0.0191101	0.0125925	0.0137900	0.0137900	0.0129347
3*p*	0.025	0.0437587	0.0436848	0.0437072	0.0437069	0.0437069	0.0437066
	0.050	0.0333681	0.0332390	0.0331623	0.0331645	0.0331645	0.0331602
	0.075	0.0243837	0.0242183	0.0239207	0.0239397	0.0239397	0.0239173
	0.100	0.0168056	0.0166227	0.0159825	0.0160537	0.0160537	0.0159798
	0.150	0.0058680	0.0057067	0.0040162	0.004463	0.0044663	0.0040316
3*d*	0.025	0.0437587	0.0435371	0.0436044	0.0436030	0.0436030	0.0436028
	0.050	0.0333681	0.0329817	0.0327508	0.0327532	0.0327532	0.0327495
	0.075	0.0243837	0.0238893	0.0229948	0.0230307	0.0230307	0.0230109
	0.100	0.0168056	0.0162600	0.0143364	0.0144842	0.0144842	0.0144147
4*p*	0.025	0.0200000	0.0199625	0.0199486	0.0199489	0.0199489	0.019948
	0.050	0.0112500	0.0111938	0.0110442	0.0110582	0.0110582	0.0110422
	0.075	0.0050000	0.0049439	0.0045370	0.0046219	0.0046219	0.0045340
	0.100	0.0012500	0.0012128	0.0004269	0.0007549	0.000755	0.0004252
4*d*	0.025	0.02000000	0.0198877	0.0198457	0.0198463	0.0198462	0.0198444
	0.050	0.0112500	0.0110819	0.0106327	0.0106674	0.0106674	0.0106355
	0.075	0.0050000	0.0048327	0.0036111	0.0038345	0.0038345	0.0036479
4*f*	0.025	0.0200000	0.0197756	0.0196914	0.0196911	0.0196911	0.0196903
	0.050	0.0112500	0.0109150	0.0100154	0.0100620	0.0100620	0.0100463
	0.075	0.0050000	0.0046682	0.0022222	0.0025563	0.0025563	0.0024452
5*p*	0.025	0.00945312	0.0094325	0.0094017	0.0094036	0.0094036	0.0094011
	0.050	0.00281250	0.0027900	0.0026067	0.0026490	0.0026490	0.0026047

## Conclusion

In this research work, we obtained analytical solutions of Dirac equation for spin and pseudospin symmetries with hyperbolic Hulthen plus hyperbolic exponential inversely quadratic potential within the framework of parametric Nikiforov–Uvarov method. Using Maple software package, numerical solutions for both the spin and pseudospin symmetries were obtain. The nonrelativistic energy equation were obtain by applying nonrelativistic limit to the relativistic energy equation. The Partition function and other thermodynamic properties were obtain using the nonrelativistic energy equation presented in a close form. The proposed potential reduces to Hulthen and exponential inversely quadratic potential as special cases. The numerical results obtained for Hulthen potential, spin and pseudospin bound state energies and thermodynamic plots are in agreement to the work of other researchers.
